# A novel telomere-related gene prognostic signature for survival and drug treatment efficiency prediction in lung adenocarcinoma

**DOI:** 10.18632/aging.204877

**Published:** 2023-08-16

**Authors:** Haiming Chen, Weiquan Liang, Weiqiang Zheng, Feilong Li, Xingxi Pan, Yiyu Lu

**Affiliations:** 1 Department of Oncology, The Sixth Affiliated Hospital, School of Medicine, South China University of Technology, Foshan, Guangdong Province 528200, China; 2 Department of Respiration, Foshan Second People's Hospital, Foshan, Guangdong Province 528000, China

**Keywords:** telomere, telomere-related gene, prognosis, lung adenocarcinoma

## Abstract

Objective: Telomere-related genes (TRGs) play a critical role in various types of tumors. However, there is a lack of comprehensive exploration of their relevance in lung cancer. This research aimed to verify the relationship between TRGs gene expression and the prognosis of patients with lung adenocarcinoma (LUAD), as well as the prediction of drug treatment efficiency.

Methods: A total of 2093 TRGs were acquired from TelNet. The clinical information including age, tumor stage, follow up and outcome (death/survival) and TRGs expression profile of LUAD were obtained from the patients in The Cancer Genome Atlas (TCGA) database and the Clinical Proteomic Tumor Analysis Consortium (CPTAC) database. The two databases were used to construct and verify a prognostic model based on the expression of hubTRGs. The tumor mutation burden, immune infiltration and subtypes, as well as IC50 prediction of multiple targeted drugs were also evaluated in TRGs-divided risk groups.

Results: A total of 335 TRGs were significantly differentially expressed in LUAD as compared with normal control. Among them, 9 TRGs (ABCC2, ABCC8, ALDH2, FOXP3, GNMT, JSRP1, MACF1, PLCD3, SULT4A1) were finally identified as hubGenes and used to construct a TRG risk score. The TRG risk score showed favorable performance in constructing a prognostic nomogram in predicting survival of LUAD, and the ROC curves at 1, 3 and 5 years were plotted and the AUROC values were 0.743, 0.754 and 0.735, respectively. Higher TRGs risk score correlated with worse immune subtypes and higher tumor mutation burden in LUAD tissues. In addition, the patients in TRG high risk group harbored a lower TIDE score which indicated potentially better response to immunotherapy.

Conclusion: This study proposed a broad molecular signature of telomere-related genes that can be used in further functional and therapeutic investigations, and also represents an integrated modality for characterizing critical molecules when exploring novel targets for lung cancer immunotherapy.

## INTRODUCTION

Lung cancer is the most common type of cancer worldwide, and lung adenocarcinoma (LUAD) is a major histologic subtype of lung cancer [1, 2]. The classic prognostic factors of LUAD are usually demographic factors such as age, complication and tumor-related parameters including tumor stage, node stage and metastasis stage [3, 4]. In recent years, due to the progress of sequencing technology, some models based on genetic signatures have been explored to evaluate the prognosis of LUAD patients. These models have shown a promising predictive accuracy, and a few of them might also indicate the potential carcinogenesis mechanism of LUAD [5–7].

Telomeres are important regions at the end of chromosomes and composed of repetitive TTAGGG DNA sequences and shelterin complex [8]. In normal lung tissues, telomeres are vital for chromosome integrity, cellular replication and protection against activation of DNA damage response [9]. Telomere attrition results in cell cycle arrest and might be followed by cell division and certain disease status. Disorder of telomeres can lead to various diseases such as cardiac disease, dyskeratosis congenita and cancers [10]. Telomere length maintenance is crucial for the limitless proliferation of human cancer cells owing to the 3’-end erosion, a process intrinsic to the replication of linear chromosomes. Continual telomere shortening keeps somatic cells from aberrant proliferation and tumorigenesis by induction of senescence or apoptosis [11]. Research has reported that cancer cells with proliferative function can solve the problem of telomere shortening through a functional ribonuclease protease complex, namely telomerase [12]. The change in telomere length is related to the risk of lung cancer and may serve as a prognostic indicator for lung cancer patients [13]. However, cancer cells can circumvent this restriction by possessing a telomere maintenance mechanism (TMM) [14].

Recently, emerging studies have implied that TMM in cancer is a complicated process that is associated with hundreds of various genes [15]. These telomere-related genes (TRGs) have also shown significant relevance to the prognosis of several types of cancer. In kidney renal clear cell cancer, a risk score developed by using the expression level of TRGs is correlated with immune subtypes and tumor mutation burden, as well as can predict the outcomes of kidney cancer patients [16]. In the study by Liu et al., an 18-telomere length-related gene prognostic signature is developed in non-small cell lung cancer to access tumor immunity and predict response to PDL-1 blockade immune therapy [17]. However, they only used 168 TRGs in the analysis while the total number of TRGs that are reported to be involved in telomere maintenance has exceeded 2000 [18]. Thus, a more comprehensive analysis is needed to further understand the signature of TRGs in lung cancer.

Herein, we aim to exhaustively explore the distinct features of TRGs in LUAD. Based on the TRGs, we constructed a risk model to predict the prognosis of LUAD and then investigate the potential role of this risk model in selecting treatment agents. This study represents the systematic investigation of telomere-related molecular signatures in LUAD, which offers a deeper genetic understanding of this cancer and facilitates the development of LUAD subtype-specific therapeutic strategy.

## METHODS

### Databases and online analytical tools used in this study

The transcriptome profiling data and survival information of patients with LUAD in the training set were downloaded from The Cancer Genome Atlas (TCGA) database (TCGA-LUAD project) via the Genomic Data Commons (GDC) data portal. The tumor mutation burden (TMB) information of the TCGA-LUAD cohort were also obtained. The data of patients with LUAD in the validation set were acquired from Clinical Proteomic Tumor Analysis Consortium (CPTAC) database (CPTAC-3 project) via the GDC data portal. The protein expression level in LUAD were explored in CPTAC database and the immunohistochemical (IHC) staining image of LUAD were identified in the Human Protein Atlas (HPA) database. The estimation of immune cells infiltration in the LUAD tumor microenvironment was evaluated by selected genes analysis using CIBERSORTx [19]. The gene list of TRGs were obtained from TelNet database which offers a comprehensive compilation of more than 2000 human genes linked to telomere maintenance [18].

### Analysis of differentially expressed TRGs in TCGA-LUAD cohort

The downloaded expression profile of TCGA-LUAD were processed via normalization by the DESeq2 package in R (version 4.2.2). Principal component analysis (PCA) was performed to compare the expression profile between tumor and normal tissues. The differentially expressed TRGs between tumor and normal tissues were analysed by using the DESeq2 package in R (version 4.2.2). Differentially expressed TRGs were defined as genes with the logFC (fold change) of expression >1 or <-1 and adjusted *P* value < 0.01. The volcano plot and heatmap were illustrated to present these differentially expressed TRGs by using the ggplot2 package and pheatmap package in R (version 4.2.2), respectively.

### Identification of HubTRGs in the survival of LUAD patients and calculation of TRG risk score

To screen the TRGs that had significant relationship with LUAD prognosis, univariate analysis was performed by using Kaplan-Meier (K-M) method and univariate COX regression method. The Kaplan-Meier method compared the high-expression and low-expression of TRGs as dichotomous variables while univariate COX regression method evaluated TRGs as continuous variable. The TRGs that were overlapped in the significant genes identified by the two methods were used for further Lass regression and multivariate COX regression. The stepwise procedure was used to determine the final hubTRGs which were included in the risk score model. These survival analyses were performed using survival, survminer and glmnet packages in R.

### Construction and verification of prognostic nomogram

A prognostic nomogram to predict the 1-, 3-, and 5-year survival rates was constructed with the variables including TRG risk score, age and AJCC pathologic stage by using rms package in R (version 4.2.2). To verify the nomogram in the training set, the calibration curve and receiver operating characteristic (ROC) curve at 1-, 3-, and 5-year was plotted to estimate the accuracy of the nomogram in predicting the prognosis by using rms, pROC and timeROC packages in R (version 4.2.2). For external validation, the same analyses were also performed in the CPTAC LUAD cohort.

### TMB analysis

The tumor mutation burden was analysed in the TCGA-LUAD cohort and downloaded via the GDC data portal. The correlation between TRG risk score, expression level of hubTRGs and TMB were analysed by using the cor.test function in R (version 4.2.2). The TRG risk score, expression level of hubTRGs of each patient were divided as high level and low-level groups, and the TMB level was analysed in these groups.

### Immune characteristics evaluation and immune subtype estimation

The infiltration of the 22 types of immune cells including macrophages, CD4^+^T cells, CD8^+^T cells, B cells, ect. in the LUAD tumor microenvironment was investigated by using specific genes’ expression profile using CIBERSORTx. The relationship between 22 types of immune cells with TRG risk score was also investigated.

The immune subtypes of LUAD in the TCGA database was acquired by using the TCGAbiolinks package in R (version 4.2.2). The immune subtypes were compared in the high and low TRG risk score groups. Besides, the Tumor ImmuneDysfunction and Exclusion (TIDE) score was calculated by using an online tool provided according to the instructions.

### IC50 prediction of multiple targeted drugs

The half-maximal inhibitory concentrations (IC50) of targeted therapeutic agents were predicted using the gene expression level to reflect the treatment sensitivity. This was performed using the oncoPredict package in R (version 4.2.2) [20].

### Statistical analysis

The analysis in this study was performed using R (version 4.2.2). The distribution normality for continuous variables was evaluated by Shapiro-Wilk test. If the continuous variables were normally distributed, they were compared by student’s t-test; otherwise, Wilcoxon ranked-sum test was conducted. *P* < 0.05 (two sides) was considered as statistically significant.

### Data availability

All data generated or analysed during this study are included in this article and its supplementary material files. Further enquiries can be directed to the corresponding author.

## RESULTS

### Differentially expressed TRGs in LUAD

**Figure 1:**
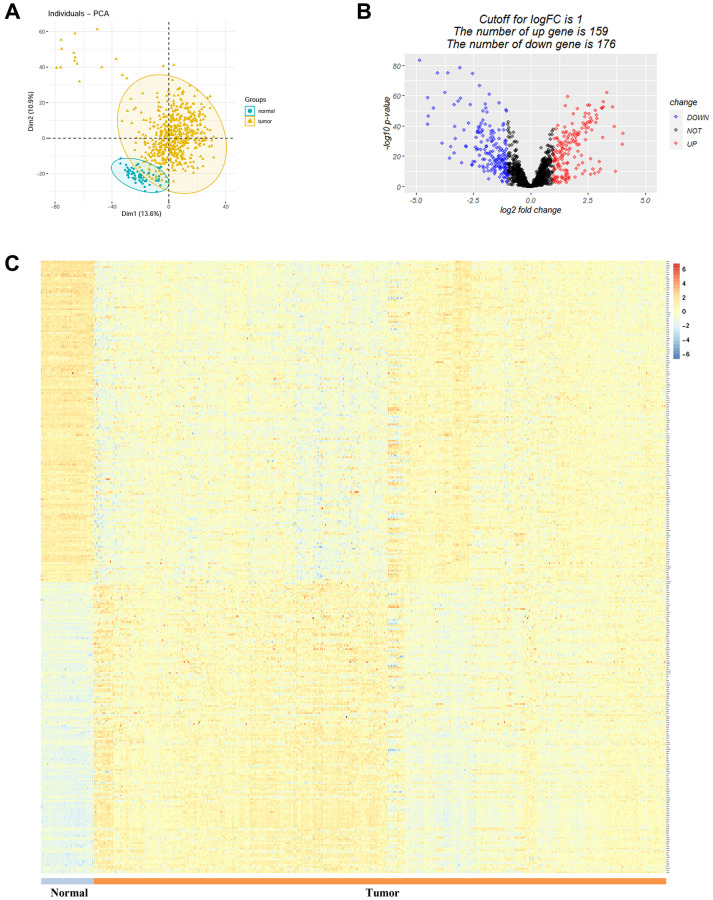
**Analysis of differentially expressed telomere-related genes in LUAD.** (**A**) Principal component analysis between LUAD and normal control. (**B**) and (**C**) Volcano plot and heatmap showing the up-regulated and down-regulated telomere-related genes in LUAD.

A total of 2093 TRGs were acquired from TelNet. The expression of these genes was compared between TCGA-LUAD tumor tissues and normal controls. PCA plot showed discrete scatter points of the two groups (Figure 1A). Among the 2093 TRGs, 335 were significantly differentially expressed in LUAD (|logFC| >1 and adjusted *P* value < 0.01). Among these, 159 were up-regulated and 176 were down-regulated (Figure 1B). The detailed expression information of the 2093 TRGs were available in Supplementary Table 1 and Figure 1C.

### Construction and verification of TRG risk model in predicting the prognosis of LUAD patients

**Figure 2:**
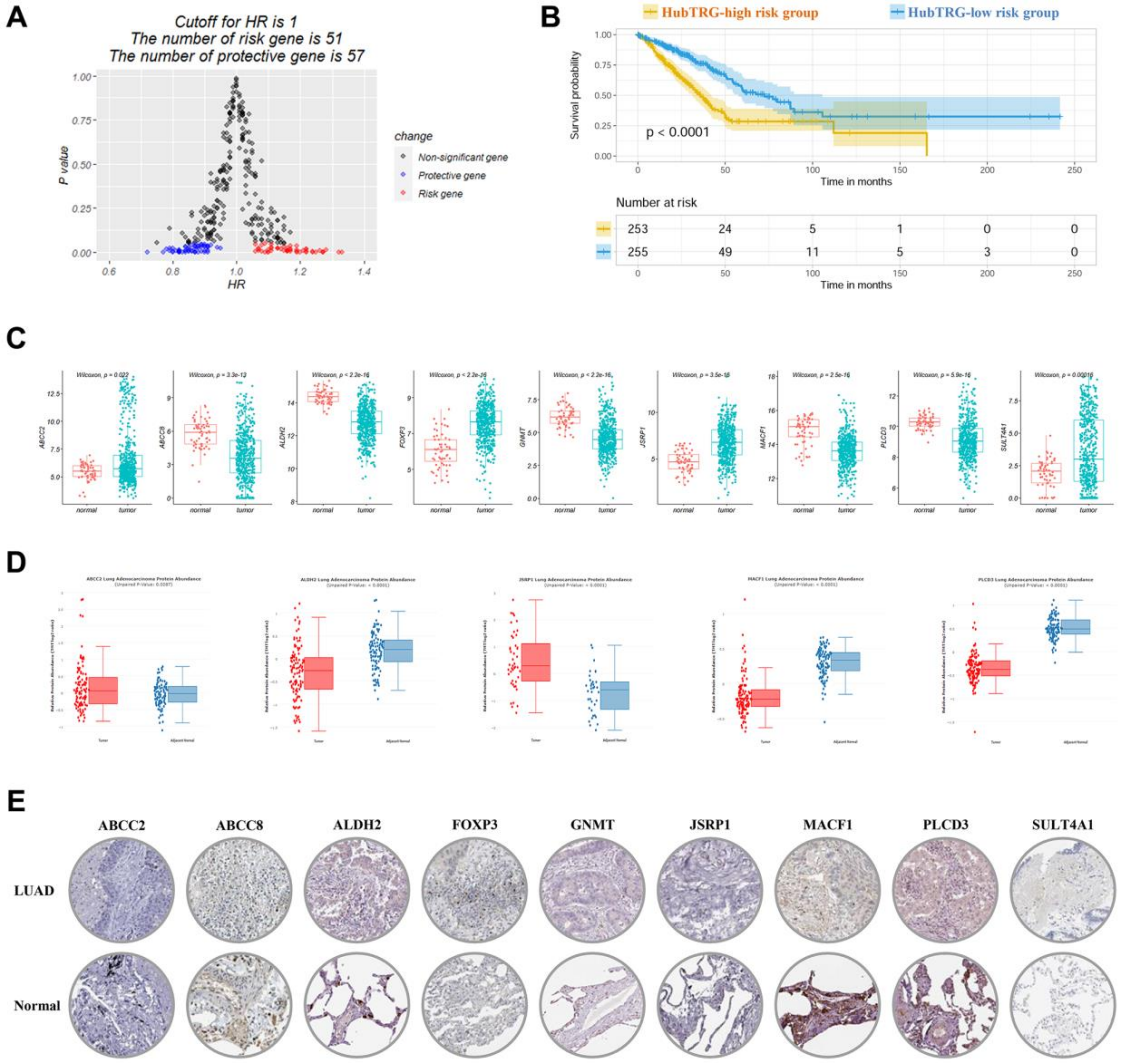
**Risk score calculated by using nine hub-telomere-related genes (TRGs) in LUAD.** (**A**) Volcano plot showing the significant genes and hazard ratio values in univariate COX regression analysis. (**B**) Survival curves of high and low TRG risk score groups plotted by K-M method. (**C**) Boxplots of nine hubTRGs mRNA expression in LUAD. (**D**) and (**E**) Boxplots and immunohistochemistry staining of the nine hub-telomere-related proteins in LUAD and normal control.

As shown in Supplementary Table 2, K-M method revealed 86 TRGs that were correlated with survival and univariate COX regression revealed 108 TRGs with *P* < 0.05. Among the 108 TRGs, 51 were risk genes with HR > 1 and 57 were protective with HR < 1 (Figure 2A). There were 74 genes that were overlapped in the results of the two methods. Then 19 out of 74 genes were screened by using Lasso regression. With further stepwise selection, a total of 9 hubTRGs were finally identified (Table 1). Using multivariate COX regression analysis, the TRG risk score were calculated with the following formula: ABCC2 × 0.0411 – ABCC8 × 0.0785 – ALDH2 × 0.1519 – FOXP3 × 0.1557 – GNMT × 0.1039 – JSRP1 × 0.0862 – MACF1 × 0.214 + PLCD3 × 0.1535 – SULT4A1 × 0.0562. The TRG risk score of patients in the TCGA-LUAD cohort was computed, and the patients were divided into high and low risk groups based on each patient’s median TRG risk score value. The K-M curve was plotted and shown in Figure 2B. The hubTRG-high risk group had significantly worse survival outcome as compared with low risk group (median survival: 35.8 versus 72.5 months, *P* < 0.0001). The mRNA expression and protein levels were shown in Figure 2C–2E.

**Table 1:** Parameters of the TRG risk score calculation.

**TRG**	**LogFC in LUAD**	**Co.ef in the multivariable** **COX model**	**Standard error of** **Co.ef**	***P* value of TRG in the** **multivariable COX model**
ABCC2	1.18	0.0411	0.028	0.142
ABCC8	−2.02	−0.0785	0.0377	0.037
ALDH2	−1.59	−0.1519	0.0742	0.041
FOXP3	1.49	−0.1557	0.0805	0.053
GNMT	−1.61	−0.1039	0.0648	0.109
JSRP1	2.09	−0.0862	0.0438	0.049
MACF1	−1.16	−0.2140	0.098	0.029
PLCD3	−1.13	0.1535	0.0669	0.022
SULT4A1	1.67	−0.0562	0.031	0.071

Abbreviations: TRG: telomere-related gene; LUAD: lung adenocarcinoma.

**Figure 3:**
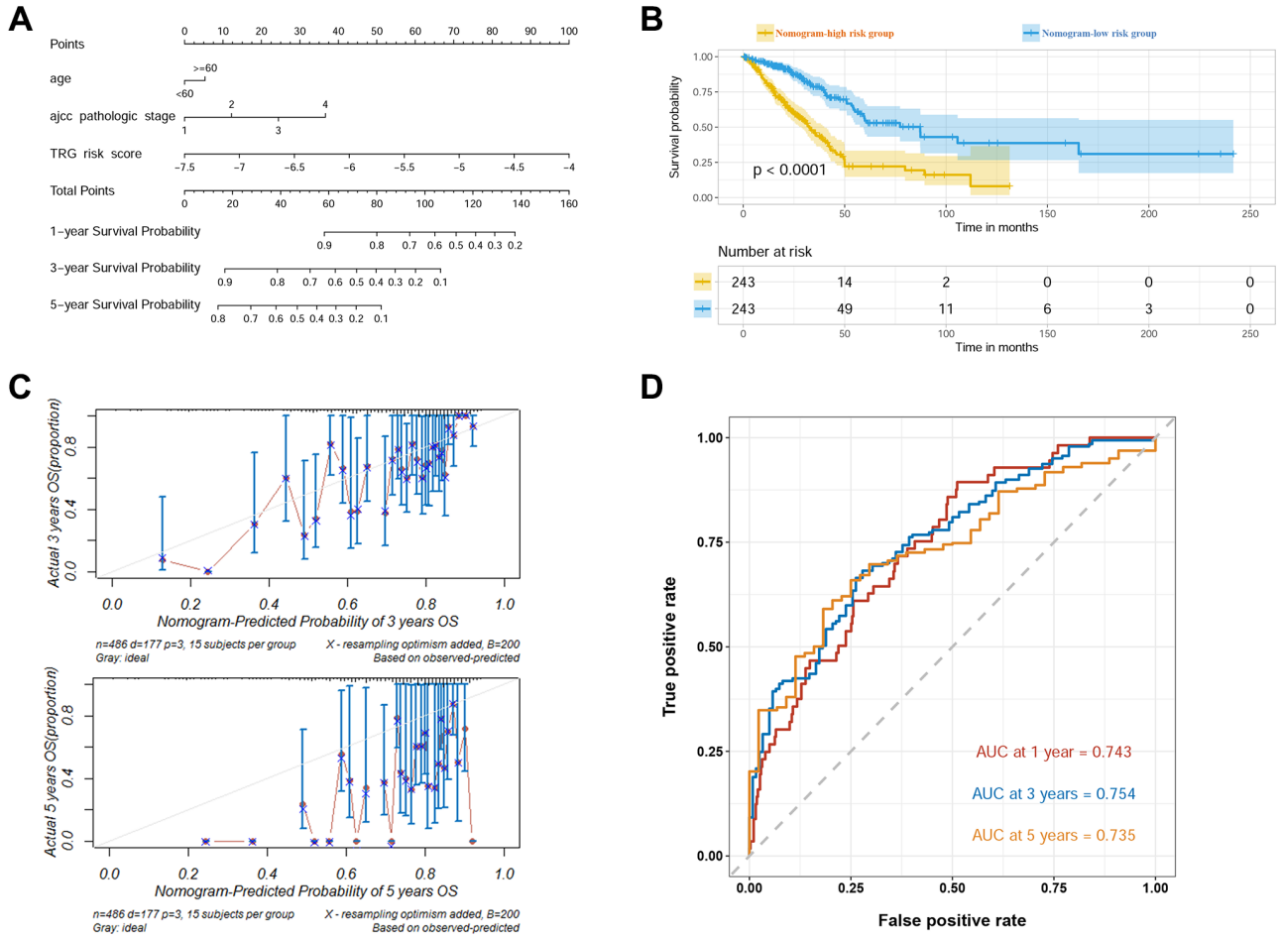
**Construction of the prognostic nomogram in TCGA cohort.** (**A**) Visualized nomogram based on age, AJCC stage, and TRG risk score. (**B**) Survival curves of high and low nomogram risk groups plotted by K-M method. (**C**) Calibration curves of the nomogram at 3 and 5 years. (**D**) ROC curves of the nomogram at 1, 3 and 5 years.

As the final TRG risk model, a nomogram was constructed based on the TRG risk score, age and AJCC pathologic stage (Figure 3A). In the nomogram, the coefficiency values of TRG risk score, age and AJCC pathologic stage were 0.9816, 0.1828 and 0.4197, respectively. Based on the nomogram, the patients in the TCGA-LUAD cohort were divided into high and low risk groups and K-M curve revealed significantly reduced survival in high risk group (Figure 3B). The calibration curves showed good fitting of predicted survival and actual survival at 3 years and 5 years (Figure 3C). The ROC curves at 1, 3 and 5 years were plotted and the AUROC values were 0.743, 0.754 and 0.735, respectively (Figure 3D).

**Figure 4:**
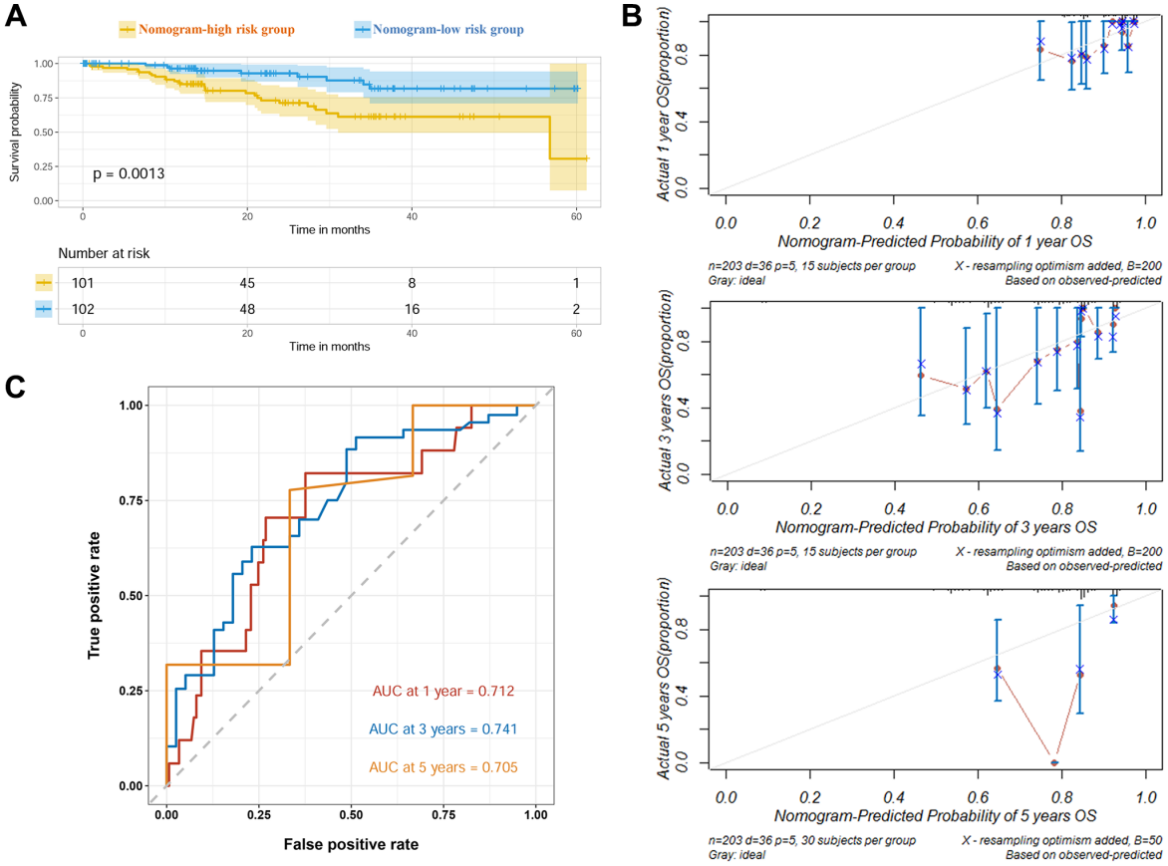
**Verification of the prognostic nomogram in CPTAC cohort.** (**A**) Survival curves of high and low nomogram risk groups plotted by K-M method. (**B**) Calibration curves of the nomogram at 1, 3 and 5 years. (**C**) ROC curves of the nomogram at 1, 3 and 5 years.

To further evaluate the accuracy of the nomogram, it was validated in an external CPTAC-LUAD cohort. The nomogram-divided risk groups showed significantly different survival within 60 months follow-up (Figure 4A). The calibration curves showed fitting of predicted survival and actual survival at 1 year, 3 years and 5 years (Figure 4B). The AUROC values at 1, 3 and 5 years were 0.712, 0.741 and 0.705, respectively (Figure 4C). In brief, the nomogram based on TRG risk score performed well in predicting prognosis of LUAD patients in both the training set and validation set.

### Patients with high TRG risk score showed higher TMB

**Figure 5:**
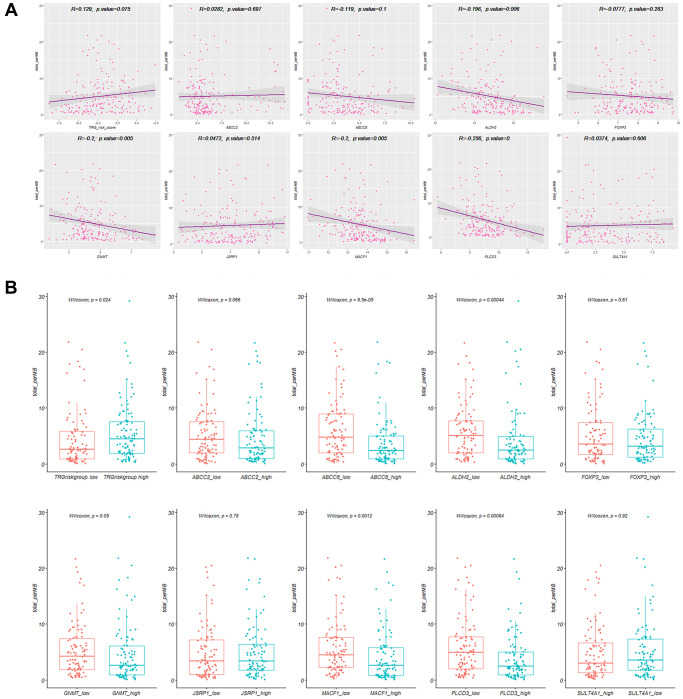
**Tumor mutation burden (TMB) correlation with TRGs.** (**A**) The correlation plots of TMB with TRG risk score and nine hub TRGs. (**B**) Level of TMB in high and low TRG risk score groups and nine hub TRGs expression.

The TRG risk score showed a positive correlation with the TMB (R = 0.129, *P* = 0.075, Figure 5A). The patients in the high TRG risk score group showed a higher TMB (*P* = 0.024) than those in the low risk group (Figure 5B). The correlation of 9 hubTRGs with TMB were also presented in Figure 5.

### TRG risk score indicated different immune cells infiltration status

**Figure 6:**
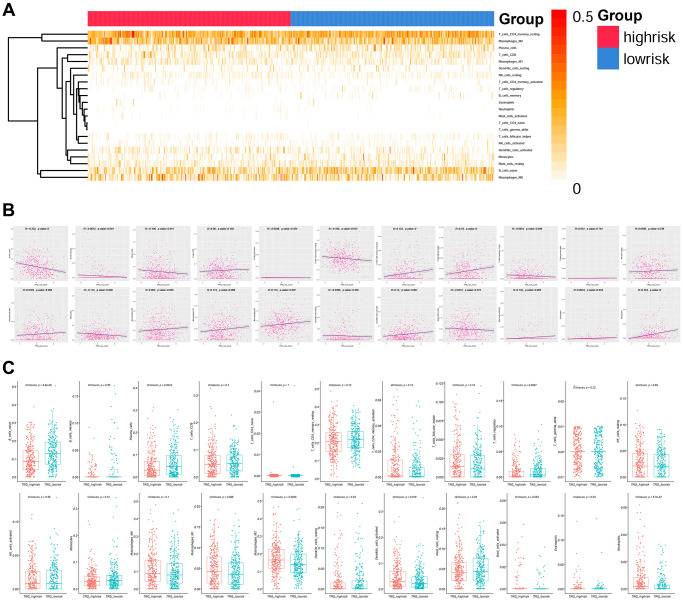
**Immune infiltration analysis.** (**A**) Heatmap showing the infiltration percentage of 22 types of immune cells in high and low TRG risk score groups. (**B**) and (**C**) Correlation of the infiltration percentage of 22 types of immune cells with TRG risk score.

The infiltration status of 22 types of immune cells was evaluated high and low TRG risk groups (Figure 6A). The infiltration of 14 types of immune cells were significantly correlated with TRG risk score (*P* < 0.05, Figure 6B), while 7 types of immune cells showed significant difference between high and low TRG risk groups (*P* < 0.05, Figure 6C).

**Figure 7:**
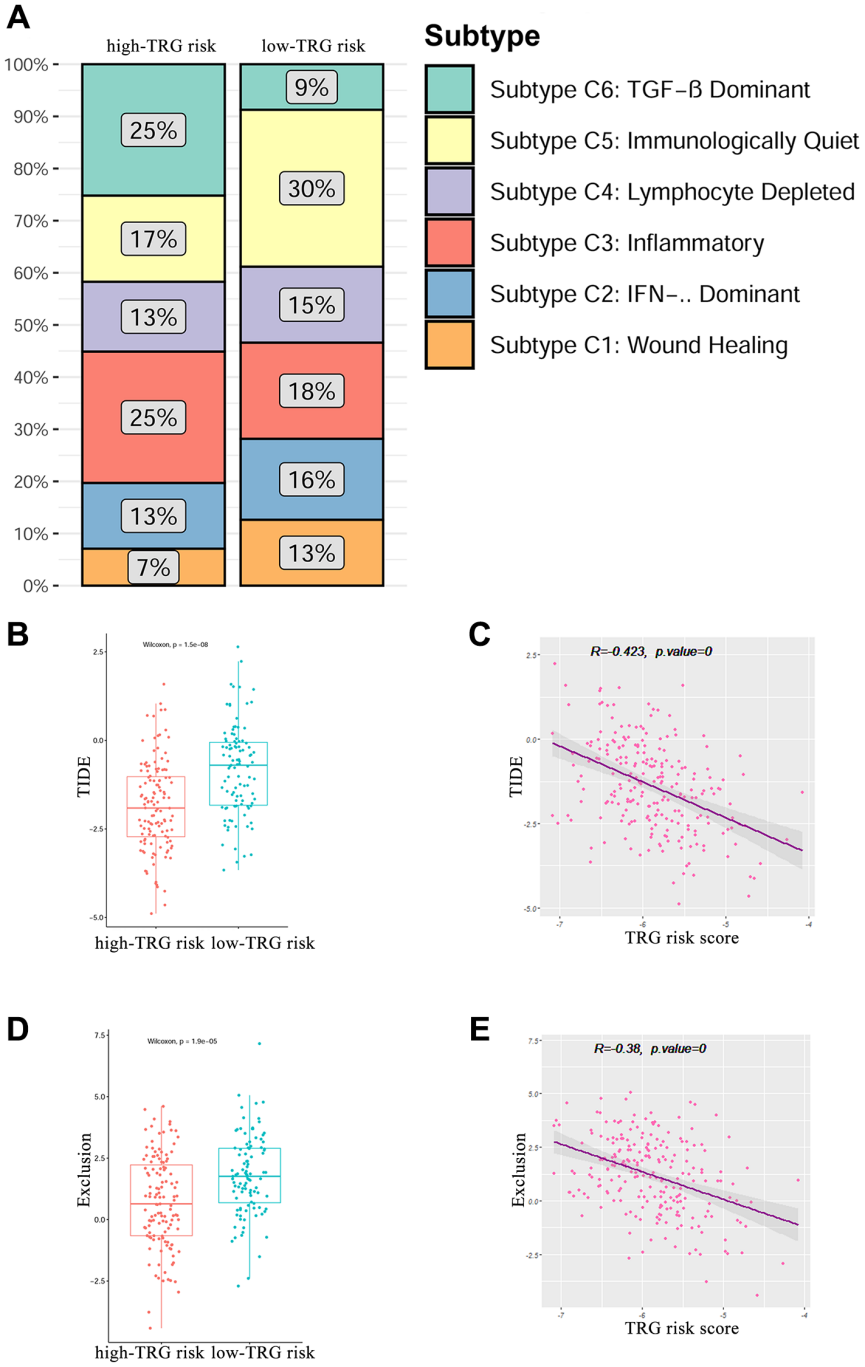
**Immune subtypes of the TRGs risk group.** (**A**) Percentage of the six immune subtypes in the high and low TRGs risk groups. (**B**) and (**C**) Correlation of the TIDE score with TRG risk score. (**D**) and (**E**) Correlation of the exclusion score with TRG risk score.

In previous research, the cancers of each individual in the TCGA database have been clustered into six subtypes based on the immune status: C1 (wound healing), C2 (IFN-g dominant), C3 (inflammatory), C4 (lymphocyte depleted), C5 (immunologically quiet), and C6 (TGF-b dominant) [21]. We analysed the immune subtypes in high and low TRG risk groups. The results showed that high TRG risk group had a higher proportion of C3 (25%) and C6 (25%) subtypes and a lower proportion of C1 (7%), C2 (13%), C4 (13%) and C5 (17%) subtypes than the patients in the low risk group (*P* = 0.005, Figure 7A).

The TIDE score was significantly lower in the TRG high risk group than in the low-risk group (*P* < 0.001, Figure 7B). The TIDE score showed significantly negative correlation with TRG risk score (R = –0.423, *P* < 0.001, Figure 7C). Similarly, the exclusion score was significantly lower in the TRG high risk group than in the low-risk group (*P* < 0.001, Figure 7D). The TIDE score was significantly negative correlated with TRG risk score (R = -0.38, *P* < 0.001, Figure 7E).

### TRG risk score in choosing treatment strategy

**Figure 8:**
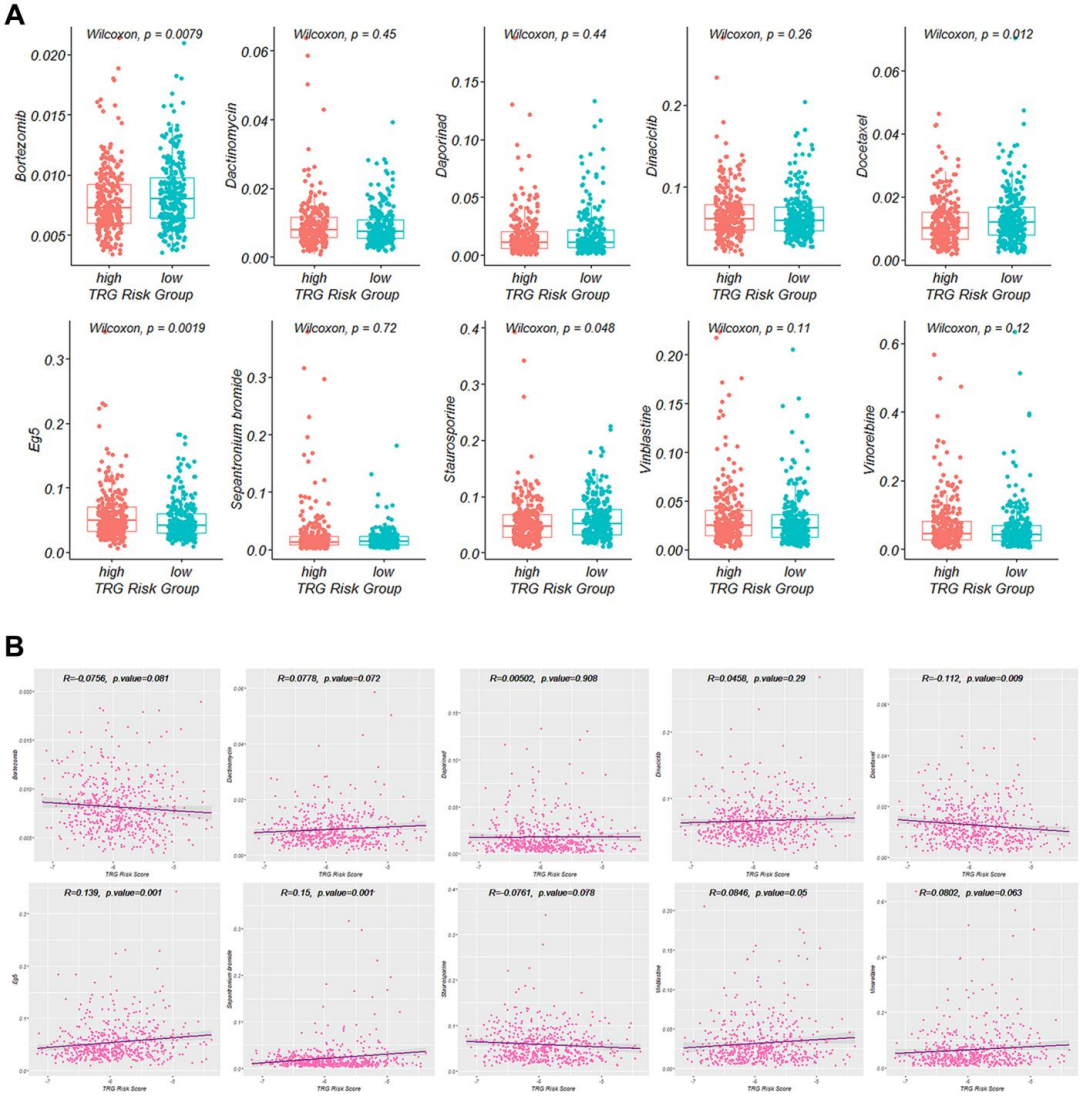
**Targeted agents’ treatment sensitivity.** (**A**) and (**B**) Correlation of the IC50 value of the selected ten drugs with TRG risk score.

The top ten drugs with the least IC50 values were identified: Bortezomib, Dactinomycin, Docetaxel, Daporinad, Sepantronium bromide, Vinblastine, Eg5, Staurosporine, Vinorelbine and Dinaciclib. Among these drugs, the IC50 values of Bortezomib, Docetaxel and Staurosporine in the high TRG risk group were significantly lower than in the low risk group, while Eg5 had a significantly higher IC50 value in the high TRG risk group (Figure 8).

## DISCUSSION

TRGs play a critical role in various types of tumors. However, there is a lack of comprehensive exploration of their relevance in lung cancer [22–25], especially in LUAD. This research aimed to verify the relationship between TRGs gene expression and the prognosis of patients with LUAD, as well as the prediction of drug treatment efficiency. To the best of our understanding, this study is the first to explore the hubGenes among more than 2000 TRGs in the prognosis of LUAD. We established a prognostic nomogram based on TRGs using public databases. We found that the TRG risk score can serve as a potential tool for selecting therapeutic drugs for LUAD.

C6 (TGF-β Dominant) subtype usually account for a small group and do not dominant in major TCGA subtypes [16]. However, in this study the C6 subtype account for a quarter of all subtypes in high TRG risk group and outnumbered the lower risk group. C6 was found to display the highest TGF-β signature and a high lymphocytic infiltration with an even amount of type I and type II T cells [21]. As compared with other subtypes, C6 specifically showed an enrichment in *KRAS* G12 mutations. C6 subtype was reported to confer the worst favorable outcome in its constituent tumors, and showed complex features reflecting a macrophage dominated, low lymphocyte infiltration, with high M2/M1 macrophage ratio, consistent with an immunosuppressed tumor microenvironment for which a poor outcome would be expected [21]. The immune subtype analysis indicated that the poor prognosis of patients in the TRG high risk group might be due to the higher portion of C6 subtype.

A higher TMB in tumor is usually considered as a biomarker to estimate the potential benefit of immune checkpoint blockade therapy. Our study showed that the TRG high risk group in LUAD was associated with a significantly higher TMB. Besides, the TRG high risk group harbored a lower TIDE score indicating that these patients might have relatively better response to immunotherapy. The drug sensitivity estimation analysis results suggested that the high-risk group patients might be more sensitive to Bortezomib, Docetaxel and Staurosporine. On the other hand, patients in the TRG low risk group might benefit from Eg5 treatment. In brief, the patients with a high TRG risk score might be more suitable candidates to receive immune checkpoint inhibitors and had less limited selection of treatment.

The genes in the TRG risk score have been reported to play multiple roles in disease especially cancers. ABCC2 is a multidrug resistance-associated protein in cancer and associated with resistance to cisplatin. The knockdown ABCC2 could reverse cisplatin resistance in lung cancer cells [26]. And ABCC8 is closely related to immune infiltration of macrophages M2. Studies have shown that knocking out ABCC8 in mice can reduce the infiltration of pro-inflammatory macrophages [27], while promoting the macrophage M2 phenotype (CD163) over the M1 phenotype (CD86) [28]. ALDH2 polymorphisms is found to be related to cancer occurrence and development. Besides, ALDH2 is a biomarker of cancer stem cells (CSCs) and is associated with proliferation, invasiveness, and multidrug resistance to chemotherapy reagents [29]. Recently, the MACF1 mutation was found to be a potential prognostic biomarker and therapeutic target for breast cancer. Patients with MACF1 mutation harbored a poor prognosis and higher tumor mutation burden score. MACF1 mutation was also found to upregulate the mTOR signaling pathway and change tumor immune microenvironment [30]. In a word, the hubTRGs can participate in the tumorigenesis, cancer cell proliferation and metastasis in various pathways.

This study has several limitations. Firstly, despite that we used a large TCGA database to construct the prognostic model and another database for external validation, we cannot perform an independent clinical trial to verify these findings due to the long duration of translation and follow-up and the high cost. Secondly, since the analysis in this study was performed by using transcriptomics data, which might limit the clinical promotion of the TRGs risk model, and a further simple and convenient method should be developed. Thirdly, we were unable to verify these findings under different genetic backgrounds due to the lack of racial data, which requires further research in the future. Lastly, more basic experiments are needed to further clarify the functions and mechanisms of the 9 hubTRGs in LUAD.

## CONCLUSION

In this study, we have constructed and validated a risk model based on telomere-related genes in lung adenocarcinoma using the two databases. The TRG signature we have identified might be of help in the selection of treatment reagents for lung adenocarcinoma patients.

## Supplementary Materials

Supplementary Tables
