# Supplementary material for: A novel telomere-related gene prognostic signature for survival and drug treatment efficiency prediction in lung adenocarcinoma
Source: Aging (Albany NY). 2023 Aug 16;15(16):7956–73. doi: 10.18632/aging.204877 (PMC10497012; doi:10.18632/aging.204877)
Supplement: Supplementary Tables [file aging-15-204877-s001.pdf]

SUPPLEMENTARY TABLES

Supplementary Table 1. Differentially expressed telomere-related gene in LUAD.

TRG	logFC	*P* value	TRG	logFC	*P* value	TRG	logFC	*P* value	TRG	logFC	*P* value
**ANGPT4**	−4.85	5.03E-84	**KLF2**	−2.13	8.47E-37	**TDRD10**	−1.58	6.32E-09	**JUNB**	−1.18	1.33E-18
**TNNC1**	−4.5	3.40E-59	**GATA2**	−2.11	5.10E-38	**AR**	−1.58	9.09E-13	**CSRP1**	−1.18	2.97E-42
**ANXA8L1**	−4.49	1.01E-41	**ALDH1A1**	−2.09	1.12E-18	**USP2**	−1.58	6.29E-17	**MYO1C**	−1.18	4.83E-38
**SH3GL2**	−4.47	3.47E-47	**PRKCQ**	−2.09	4.06E-34	**DYDC1**	−1.57	5.96E-14	**MACF1**	−1.16	6.75E-17
**GPA33**	−4.24	1.66E-52	**LGALSL**	−2.09	1.32E-44	**PHYHD1**	−1.56	2.66E-11	**TXNIP**	−1.16	2.84E-14
**FHL1**	−4.08	8.23E-76	**PLCL1**	−2.08	1.57E-50	**ELOVL3**	−1.56	2.94E-10	**ABCC6**	−1.16	3.18E-10
**FOSB**	−3.88	2.17E-29	**PNMT**	−2.08	3.09E-14	**SYNE1**	−1.52	9.90E-18	**SYDE1**	−1.15	1.88E-22
**PTPN5**	−3.75	1.21E-62	**DAW1**	−2.05	5.00E-13	**CEBPA**	−1.51	3.69E-14	**EPHX2**	−1.15	6.48E-13
**PRX**	−3.63	9.87E-76	**ABCC8**	−2.02	1.56E-10	**DST**	−1.5	1.40E-15	**FOXO1**	−1.15	3.96E-19
**XAGE2**	−3.55	1.49E-19	**SULT1B1**	−2.01	9.92E-19	**ABCC9**	−1.5	3.51E-18	**SMARCA2**	−1.14	4.56E-24
**ZBTB16**	−3.48	5.48E-27	**LRCH2**	−2	4.94E-25	**MITF**	−1.5	6.03E-29	**TNIP3**	−1.13	2.64E-06
**PCSK9**	−3.34	5.70E-23	**WFS1**	−1.97	4.31E-55	**ARRDC4**	−1.48	3.63E-26	**PLCD3**	−1.13	3.65E-11
**ANOS1**	−3.34	5.83E-55	**PDE1B**	−1.97	5.25E-42	**DMD**	−1.44	7.69E-17	**RAPGEF3**	−1.13	4.80E-15
**FGFR4**	−3.32	3.53E-40	**KLF6**	−1.97	1.16E-52	**SYDE2**	−1.42	1.10E-18	**FOXO4**	−1.12	1.73E-29
**LRRC18**	−3.28	3.47E-32	**EGR1**	−1.96	9.33E-23	**LRRC25**	−1.41	1.48E-20	**TNIK**	−1.12	8.90E-10
**IQSEC3**	−3.17	4.21E-57	**PPP1R17**	−1.94	5.38E-27	**HOXA7**	−1.4	3.25E-14	**LRCH1**	−1.1	3.99E-31
**TAL1**	−3.08	5.44E-79	**AHNAK**	−1.92	2.98E-27	**PRKD1**	−1.4	2.74E-19	**BIN2**	−1.1	3.89E-16
**TUBB1**	−3.07	4.82E-59	**FLI1**	−1.91	5.53E-50	**VAMP2**	−1.39	7.02E-38	**ITGAX**	−1.1	8.85E-13
**WDR38**	−3	1.96E-16	**S100A8**	−1.9	4.01E-13	**ETS1**	−1.39	8.29E-27	**MSN**	−1.09	2.19E-20
**PDK4**	−2.91	6.85E-38	**FBP1**	−1.89	2.95E-31	**PPM1F**	−1.38	5.01E-56	**NR2F1**	−1.09	3.81E-13
**CLIC3**	−2.84	1.83E-27	**CDK15**	−1.87	2.10E-22	**RARRES2**	−1.38	3.72E-16	**NEK7**	−1.09	1.01E-30
**MYOCD**	−2.81	5.94E-27	**NLGN4X**	−1.86	2.34E-15	**TFRC**	−1.38	2.93E-21	**SLC7A8**	−1.07	9.03E-09
**KLF4**	−2.7	1.44E-42	**PRDM16**	−1.86	3.60E-11	**KAT2B**	−1.38	1.73E-32	**PRKCB**	−1.07	1.57E-11
**SNX22**	−2.63	1.24E-54	**ZFP42**	−1.82	1.50E-05	**NCBP2L**	−1.36	3.87E-13	**PAPSS1**	-1.07	2.58E-51
**CFTR**	−2.57	8.81E-15	**ADPRH**	−1.8	1.32E-61	**FGFR3**	−1.36	8.27E-07	**TLN1**	−1.07	1.04E-28
**ASPG**	−2.57	4.72E-15	**CDKN2B**	−1.8	2.27E-22	**ARHGAP23**	−1.35	2.04E-24	**OSBPL11**	−1.06	9.54E-51
**EPAS1**	−2.54	1.74E-75	**KLF9**	−1.79	9.71E-41	**TFEC**	−1.34	3.79E-14	**FOXN3**	−1.06	1.89E-25
**ALPL**	−2.5	3.00E-17	**FOS**	−1.79	9.86E-18	**CHD5**	−1.34	4.18E-12	**HHATL**	−1.06	0.001297
**MACROD2**	−2.43	3.60E-17	**FOXF2**	−1.76	1.08E-23	**PALM**	−1.33	4.47E-17	**PLCL2**	−1.05	1.88E-15
**CFAP58**	−2.41	8.33E-27	**TCEAL7**	−1.75	1.23E-32	**LRRC63**	−1.29	4.08E-13	**ARHGAP20**	−1.05	2.34E-06
**KCTD16**	−2.37	3.92E-33	**MYH10**	−1.73	3.90E-37	**PTPRG**	−1.29	6.74E-19	**MECOM**	−1.05	1.12E-08
**PAK5**	−2.35	5.93E-25	**SLC7A7**	−1.73	2.16E-35	**THEMIS2**	−1.28	1.89E-25	**JAK2**	−1.04	6.56E-19
**GATA6**	−2.33	2.44E-36	**ETV1**	−1.73	2.90E-21	**FERMT3**	−1.28	1.19E-18	**BMP7**	−1.04	0.0004118
**SOX5**	−2.31	9.75E-38	**TDRD9**	−1.72	4.36E-11	**JUN**	−1.27	2.13E-20	**JAZF1**	−1.04	2.08E-21
**GATA5**	−2.3	1.67E-25	**RYR2**	−1.72	1.72E-15	**IRS4**	−1.27	7.17E-09	**CREB5**	−1.02	1.26E-08
**SIRPD**	−2.28	3.86E-40	**ZEB2**	−1.7	2.96E-30	**IP6K3**	−1.26	0.0001382	**TRIM22**	−1.02	1.54E-12
**KLF17**	−2.27	1.97E-36	**PLCB4**	−1.69	5.54E-12	**ARL11**	−1.26	8.13E-18	**MT1X**	−1.01	1.76E-07
**DOK2**	−2.24	2.48E-39	**PLCE1**	−1.68	1.12E-30	**DOCK2**	−1.25	5.16E-16	**ARHGEF28**	−1	3.55E-08
**PDLIM2**	−2.23	3.11E-67	**ARRB1**	−1.67	8.46E-38	**NFATC1**	−1.24	2.24E-24	**BAIAP2L1**	1	4.61E-23
**DYDC2**	−2.22	6.85E-14	**AK1**	−1.66	4.66E-24	**GABRB3**	−1.23	0.0007302	**BASP1**	1.01	1.67E-07
**KLF15**	−2.22	2.31E-21	**TUBB6**	−1.66	2.60E-33	**CDK14**	−1.22	1.01E-17	**GMNN**	1.01	3.55E-21
**GATA1**	−2.22	4.06E-36	**SLC6A12**	−1.65	3.53E-20	**MAP3K3**	−1.21	2.95E-41	**GLDC**	1.01	0.0001704
**CAMK2A**	−2.21	7.08E-41	**GNMT**	-1.61	7.52E-19	**ETS2**	−1.2	1.70E-21	**PKIB**	1.02	7.91E-06
**PPARG**	−2.18	6.40E-25	**UTRN**	-1.61	5.33E-32	**GATA3**	−1.19	1.79E-13	**LRRIQ4**	1.02	2.56E-05
**TBX2**	−2.16	1.27E-42	**JUND**	−1.6	3.43E-36	**ANXA1**	−1.19	2.22E-09	**POC1A**	1.02	2.68E-17
**MYOM2**	−2.15	5.04E-30	**ALDH2**	−1.59	1.19E-27	**CDKL2**	−1.19	7.44E-07	**CHEK2**	1.02	1.07E-20
**DSG2**	1.02	5.57E-12	**PC**	1.4	5.39E-26	**RMI2**	1.87	1.37E-48	**IGF2BP1**	2.8	1.94E-09
**TFAP4**	1.03	1.74E-32	**MCM2**	1.4	4.05E-21	**ASF1B**	1.89	1.25E-37	**CENPF**	2.82	4.70E-51
**GAP43**	1.03	0.0001926	**EDN2**	1.41	5.29E-06	**CHEK1**	1.9	1.61E-35	**HMGB3**	2.84	6.90E-43
**DSCC1**	1.04	9.30E-13	**CHAF1B**	1.42	1.12E-36	**PACSIN1**	1.92	4.90E-20	**EXO1**	2.86	7.46E-48
**ZC3HAV1L**	1.05	2.30E-17	**RAD54B**	1.42	7.61E-26	**TDRD5**	1.94	8.78E-07	**AURKB**	2.89	9.61E-46
**ATAD5**	1.05	2.24E-18	**RHPN1**	1.44	5.16E-16	**AURKA**	1.96	2.31E-33	**HMGA2**	2.97	2.49E-10

**GMDS**	1.06	4.98E-19	**ATAD2**	1.45	1.02E-28	**ABCB6**	1.97	3.49E-31	**AKR7A3**	2.99	3.42E-17
**TTBK1**	1.07	1.14E-11	**FANCI**	1.47	5.84E-34	**RHBDL2**	1.98	1.41E-29	**PCP4**	3.05	2.39E-12
**RACGAP1**	1.07	6.76E-18	**CALML6**	1.47	2.81E-11	**SLC7A11**	1.99	1.84E-12	**NEK2**	3.09	1.17E-52
**TDRKH**	1.08	1.35E-24	**KAT2A**	1.48	7.00E-40	**BRIP1**	2	4.49E-36	**CDC20**	3.13	1.25E-52
**S100A7**	1.09	0.0031685	**FOXP3**	1.49	1.47E-22	**EZH2**	2.04	5.04E-52	**TFAP2A**	3.14	2.75E-33
**HRG**	1.11	5.69E-07	**FANCA**	1.51	3.76E-36	**TRIP13**	2.05	3.81E-28	**ETV4**	3.17	3.14E-53
**NME4**	1.11	1.48E-19	**RCC1**	1.51	7.06E-54	**CLSPN**	2.05	4.51E-35	**KIF4A**	3.18	1.38E-56
**SLC7A9**	1.11	2.19E-05	**BLM**	1.51	1.45E-30	**ABCC3**	2.07	7.32E-24	**TOP2A**	3.32	8.46E-63
**CCT3**	1.11	5.80E-39	**ARL14**	1.52	9.51E-05	**ORC1**	2.08	4.67E-35	**TUBB3**	3.56	2.29E-53
**RNF222**	1.12	5.33E-07	**AVPR1B**	1.53	5.07E-09	**JSRP1**	2.09	6.70E-15	**TERT**	3.66	3.62E-40
**LDHA**	1.12	7.61E-33	**HGD**	1.53	3.51E-05	**EME1**	2.1	1.89E-36	**AKR1B10**	3.7	9.98E-11
**PRDX4**	1.15	3.28E-23	**RAD51**	1.53	6.05E-25	**SLC7A5**	2.11	1.22E-28	**PITX1**	4	1.71E-28
**CCNE2**	1.15	1.06E-14	**PNCK**	1.54	5.58E-08	**RHBDL1**	2.21	1.79E-21	**GREM1**	4.03	6.81E-36
**FANCD2**	1.16	3.16E-32	**MYCN**	1.55	1.04E-06	**CCNA2**	2.25	4.07E-39			
**ABCC2**	1.18	0.0009098	**IVL**	1.55	0.0019983	**COCH**	2.27	2.45E-16			
**MCM6**	1.18	9.63E-28	**DBNDD1**	1.57	1.33E-27	**SLC7A10**	2.28	3.16E-09			
**E2F3**	1.18	5.32E-41	**LMNB1**	1.57	8.93E-37	**RECQL4**	2.28	1.34E-42			
**RUNX2**	1.18	2.67E-18	**NAT16**	1.57	1.75E-11	**IGF2BP3**	2.36	3.69E-11			
**FOXO6**	1.19	8.36E-07	**ABCC11**	1.58	6.70E-16	**HMMR**	2.36	3.07E-41			
**DNMT3B**	1.2	5.93E-15	**FOXH1**	1.6	4.03E-11	**CCNB1**	2.37	5.42E-47			
**UGDH**	1.2	2.46E-13	**TUBB8**	1.6	3.19E-16	**MAST1**	2.39	9.51E-32			
**FANCB**	1.21	6.00E-16	**PAICS**	1.61	3.36E-60	**CDCA8**	2.4	3.93E-49			
**MAGEA4**	1.23	0.0076762	**DQX1**	1.62	3.15E-09	**PKMYT1**	2.41	3.40E-39			
**FEN1**	1.25	2.41E-30	**DNA2**	1.62	1.61E-32	**CCNE1**	2.42	1.05E-36			
**WDHD1**	1.26	5.79E-24	**PIF1**	1.63	4.00E-22	**NDC80**	2.43	6.48E-42			
**MSH5**	1.28	6.19E-14	**KPNA2**	1.64	5.82E-34	**RAD54L**	2.48	5.27E-43			
**CHTF18**	1.3	9.10E-20	**POLE2**	1.64	9.46E-30	**ORC6**	2.49	2.23E-54			
**PLOD2**	1.31	1.23E-11	**PFKP**	1.66	1.08E-24	**NCAPG**	2.51	1.38E-39			
**RFC4**	1.31	5.00E-29	**UCHL1**	1.66	9.45E-07	**XRCC2**	2.55	1.26E-48			
**CALML5**	1.31	0.0007595	**TUBB2B**	1.66	5.35E-07	**PLK1**	2.56	2.38E-54			
**TXNDC17**	1.32	2.02E-22	**SULT4A1**	1.67	8.92E-06	**CDC45**	2.57	6.69E-44			
**GAPDH**	1.33	1.06E-29	**ECT2**	1.71	2.78E-35	**FOXM1**	2.6	5.06E-43			
**PDK1**	1.35	2.16E-27	**CDC25A**	1.72	7.72E-26	**S100P**	2.61	7.35E-09			
**FANCG**	1.35	8.06E-24	**PAFAH1B3**	1.75	1.31E-29	**TBX15**	2.61	6.56E-19			
**RAD51AP1**	1.35	1.69E-18	**HMGA1**	1.81	5.50E-32	**MKI67**	2.62	5.63E-46			
**RASGEF1C**	1.35	2.24E-06	**MCM4**	1.82	2.48E-42	**PSAT1**	2.68	3.60E-38			
**PAX5**	1.36	3.93E-07	**E2F2**	1.84	9.29E-35	**SGO1**	2.68	3.06E-44			
**RPS10**	1.38	1.15E-20	**CDK1**	1.86	1.66E-30	**TRIM15**	2.71	3.10E-12			

Abbreviations: TRG: telomere-related gene; LUAD: lung adenocarcinoma.

Supplementary Table 2. Significant TRGs in K-M curve and COX regression analysis.

Similar TRG in K-M	*P* value	Similar TRG in COX	Hazard ratio	*P* value	Different TRG in K-M	*P* value	Different TRG in COX	Hazard ratio	*P* value
**ABCC2**	0.007	**ABCC2**	1.1	0	**ADPRH**	0.018	**ANOS1**	0.87	0.002
**ABCC6**	0.017	**ABCC6**	0.86	0.005	**ARL11**	0.017	**ARHGAP20**	0.91	0.032
**ABCC8**	0.03	**ABCC8**	0.91	0.007	**HMGA1**	0.013	**AURKA**	1.17	0.02
**ALDH2**	0.004	**ALDH2**	0.8	0.001	**JAK2**	0.045	**BIN2**	0.82	0.008
**ARL14**	0.037	**ARL14**	1.1	0.001	**JAZF1**	0.001	**BLM**	1.22	0.018
**ARRB1**	0.048	**ARRB1**	0.77	0	**KPNA2**	0.036	**CDC25A**	1.19	0.013
**AURKB**	0.024	**AURKB**	1.11	0.047	**LDHA**	0.03	**EPAS1**	0.84	0.031
**CCNA2**	0.001	**CCNA2**	1.24	0	**PDE1B**	0.036	**ETV1**	0.9	0.037
**CCNB1**	0.01	**CCNB1**	1.22	0.003	**RASGEF1C**	0.014	**FANCI**	1.26	0.01
**CDC20**	0.022	**CDC20**	1.13	0.024	**SMARCA2**	0.019	**FOXN3**	0.78	0.02
**CDK1**	0.003	**CDK1**	1.22	0.002	**UTRN**	0.043	**GATA1**	0.89	0.042
**CDKL2**	0	**CDKL2**	0.87	0.001	**XRCC2**	0.022	**GATA2**	0.87	0.033
**CEBPA**	0.015	**CEBPA**	0.9	0.031			**HHATL**	0.9	0.009
**CENPF**	0.04	**CENPF**	1.14	0.02			**HOXA7**	1.16	0.011
**CFTR**	0.017	**CFTR**	0.91	0.002			**ITGAX**	0.85	0.017
**CHEK1**	0.003	**CHEK1**	1.27	0.001			**KLF4**	1.13	0.024
**CLSPN**	0.018	**CLSPN**	1.16	0.024			**LMNB1**	1.19	0.035
**DMD**	0.043	**DMD**	0.87	0.019			**MYOM2**	0.9	0.046
**DOCK2**	0.018	**DOCK2**	0.85	0.009			**NLGN4X**	1.09	0.048
**DOK2**	0.005	**DOK2**	0.88	0.03			**PCP4**	0.95	0.022
**DSG2**	0.032	**DSG2**	1.21	0.011			**PCSK9**	1.07	0.023
**ECT2**	0	**ECT2**	1.27	0.001			**PHYHD1**	0.91	0.026
**EPHX2**	0.049	**EPHX2**	0.88	0.036			**PITX1**	1.06	0.047
**EXO1**	0.003	**EXO1**	1.19	0.001			**PKIB**	1.11	0.026
**FBP1**	0.002	**FBP1**	0.83	0.002			**PKMYT1**	1.17	0.012
**FEN1**	0.025	**FEN1**	1.28	0.013			**PLOD2**	1.13	0.024
**FLI1**	0.022	**FLI1**	0.84	0.031			**RAPGEF3**	0.87	0.037
**FOXM1**	0.002	**FOXM1**	1.2	0.001			**RHPN1**	0.89	0.032
**FOXO4**	0.037	**FOXO4**	0.79	0.022			**SGO1**	1.16	0.007
**FOXO6**	0.007	**FOXO6**	0.91	0.035			**SLC7A11**	1.08	0.04
**FOXP3**	0.004	**FOXP3**	0.82	0.005			**SYDE2**	0.84	0.008
**GAP43**	0.02	**GAP43**	1.1	0.012			**TRIM22**	0.84	0.008
**GNMT**	0.005	**GNMT**	0.82	0.001			**TUBB6**	1.17	0.038
**HMGA2**	0.031	**HMGA2**	1.06	0.005			**WDHD1**	1.33	0.001
**HMMR**	0.001	**HMMR**	1.25	0					
**HRG**	0.006	**HRG**	1.11	0.021					
**IGF2BP1**	0.043	**IGF2BP1**	1.09	0					
**IGF2BP3**	0.032	**IGF2BP3**	1.07	0.016					
**JSRP1**	0.048	**JSRP1**	0.92	0.041					
**KAT2B**	0	**KAT2B**	0.82	0.012					
**KIF4A**	0.032	**KIF4A**	1.16	0.008					
**KLF15**	0.009	**KLF15**	0.87	0.002					
**MACF1**	0.042	**MACF1**	0.79	0.009					
**MAP3K3**	0.023	**MAP3K3**	0.72	0.001					
**MECOM**	0.03	**MECOM**	0.88	0.024					

**MKI67**	0.001	**MKI67**	1.15	0.011
**NCAPG**	0.02	**NCAPG**	1.17	0.005
**NDC80**	0.021	**NDC80**	1.19	0.004
**NEK2**	0.001	**NEK2**	1.16	0.005
**NFATC1**	0.002	**NFATC1**	0.82	0.006
**PAX5**	0.013	**PAX5**	0.89	0.005
**PDLIM2**	0.015	**PDLIM2**	0.86	0.042
**PFKP**	0.006	**PFKP**	1.15	0.03
**PLCD3**	0.014	**PLCD3**	1.32	0
**PLCL1**	0.006	**PLCL1**	0.87	0.048
**PLCL2**	0.008	**PLCL2**	0.81	0.009
**PLK1**	0.001	**PLK1**	1.27	0
**PRDM16**	0	**PRDM16**	0.9	0.002
**PRKCB**	0.006	**PRKCB**	0.82	0.001
**RACGAP1**	0.018	**RACGAP1**	1.22	0.017
**RAD51**	0.023	**RAD51**	1.24	0.003
**S100P**	0.038	**S100P**	1.07	0.007
**SLC7A8**	0.002	**SLC7A8**	0.86	0.008
**SNX22**	0.011	**SNX22**	0.82	0.001
**SULT4A1**	0.029	**SULT4A1**	0.94	0.027
**SYNE1**	0.004	**SYNE1**	0.84	0.002
**TDRD10**	0.005	**TDRD10**	0.88	0.001
**TFAP2A**	0.004	**TFAP2A**	1.11	0.007
**TFEC**	0.031	**TFEC**	0.89	0.045
**THEMIS2**	0.03	**THEMIS2**	0.8	0.006
**TNIK**	0.002	**TNIK**	0.85	0.003
**TRIM15**	0.014	**TRIM15**	1.08	0.003
**VAMP2**	0.021	**VAMP2**	0.8	0.021
**ZEB2**	0.001	**ZEB2**	0.85	0.027

Abbreviation: TRG: telomere-related gene.
